# Predicting miRNA–disease associations using improved random walk with restart and integrating multiple similarities

**DOI:** 10.1038/s41598-021-00677-w

**Published:** 2021-10-26

**Authors:** Van Tinh Nguyen, Thi Tu Kien Le, Khoat Than, Dang Hung Tran

**Affiliations:** 1 Faculty of Information Technology, Hanoi National University of Education, Hanoi, Vietnam; 2 Faculty of Information Technology, Hanoi University of Industry, 298 Cau Dien Street, Bac Tu Liem District, Hanoi, Vietnam; 3 Hanoi University of Science and Technology, Hanoi, Vietnam

**Keywords:** Computational biology and bioinformatics, Computational models, Data integration, Data mining

## Abstract

Predicting beneficial and valuable miRNA–disease associations (MDAs) by doing biological laboratory experiments is costly and time-consuming. Proposing a forceful and meaningful computational method for predicting MDAs is essential and captivated many computer scientists in recent years. In this paper, we proposed a new computational method to predict miRNA–disease associations using improved random walk with restart and integrating multiple similarities (RWRMMDA). We used a WKNKN algorithm as a pre-processing step to solve the problem of sparsity and incompletion of data to reduce the negative impact of a large number of missing associations. Two heterogeneous networks in disease and miRNA spaces were built by integrating multiple similarity networks, respectively, and different walk probabilities could be designated to each linked neighbor node of the disease or miRNA node in line with its degree in respective networks. Finally, an improve extended random walk with restart algorithm based on miRNA similarity-based and disease similarity-based heterogeneous networks was used to calculate miRNA–disease association prediction probabilities. The experiments showed that our proposed method achieved a momentous performance with Global LOOCV AUC (Area Under Roc Curve) and AUPR (Area Under Precision-Recall Curve) values of 0.9882 and 0.9066, respectively. And the best AUC and AUPR values under fivefold cross-validation of 0.9855 and 0.8642 which are proven by statistical tests, respectively. In comparison with other previous related methods, it outperformed than NTSHMDA, PMFMDA, IMCMDA and MCLPMDA methods in both AUC and AUPR values. In case studies of Breast Neoplasms, Carcinoma Hepatocellular and Stomach Neoplasms diseases, it inferred 1, 12 and 7 new associations out of top 40 predicted associated miRNAs for each disease, respectively. All of these new inferred associations have been confirmed in different databases or literatures.

## Introduction

MicroRNAs (miRNAs) are an important class of short non-coding RNAs (about 22–26 nucleotides)^1^. They play important roles in regulating many primary cellular functions such as development, differentiation, growth, signal transduction, metabolism and so on^2^. Many studies have shown that development and progression of human diseases are associated with the abnormal expression and dysregulations of the miRNAs^2,3^. Identifying miRNA–disease associations could facilitate us to understand disease mechanism at miRNA level and to detect disease biomarkers for diagnosis, treatment, prognosis, and prevention^3–6^. However, using traditional biological experimental methods to identify the associations between miRNAs and diseases is expensive and time-consuming. As more and more biological datasets be developed, it would be a forceful approach to develop computational methods to infer the latent associations between miRNAs and diseases. It has become a hot topic and captivated many computer scientists in recent years.

Recently, computational methods for predicting miRNA–disease associations have achieved extensive and prosperous applications. We could roughly divide the computational methods of miRNA–disease associations prediction into three categories as follows. Firstly, the network-based methods which are normally relied on a common assumption that miRNAs associated with diseases using similar phenotypes are similar in function, and vice versa^7^. For example, Jiang et al*.*^8^ predicted potential miRNA–disease associations by priority of disease associated miRNAs through human peptide-microRNAome. Gu et al.^9^ proposed a network consistent projection algorithm to infer latent miRNA–disease associations by integrating similarity networks and associated networks. Chen et al.^10^ proposed a computational model of Bipartite Network Projection for miRNA–disease association prediction (BNPMDA) based on the known miRNA–disease associations, integrated miRNA similarity and integrated disease similarity. Liang et al.^5^ established an Adaptive Multi-View Multi-Label model (AMVML) to learn a new affinity graph for both diseases and miRNAs to discover potential miRNA–disease associations. The main advantage of these methods is that they can be applied to predict isolated disease-associated miRNAs but their performance is not very gratifying^5^. Secondly, the machine learning methods which have been implemented to improve classification accuracy and prediction performance^4,9^. For instance, a normalized least square method (RLSMDA) was introduced by Chen and Yan^11^ to identify the potential miRNA–disease associations. Shen et al.^12^ presented the cooperative matrix decomposition (CMFMDA) algorithm in recommendation system to uncover potential associations. Xu et al.^4^ designed a probability matrix factorization model (PMFMDA) to infer potentially relevant miRNAs for disease. Chen et al.^13^ presented a model of Inductive Matrix Completion for miRNA–disease association prediction (IMCMDA). Yu et al.^14^ introduced a model named as MCLPMDA which used a matrix completion algorithm to reconstruct the new miRNA and disease matrices, and then it utilized a label propagation algorithm to predict disease-related miRNAs. Chen and Huang^15^ proposed a LRSSLMDA model to infer potential miRNA–disease associations by using sparse subspace learning with Laplacian regularization on known miRNA–disease association network and the informative feature profiles attained from integrated miRNA or disease similarity networks. Chen et al.^16^ offered a model named Neighborhood Constraint Matrix Completion for miRNA–disease Association prediction (NCMCMDA) to recover the missing miRNA–disease associations by adding similarity based neighborhood constraint into matrix completion model. Chen et al.^17^ developed a model of Decision Tree based miRNA–disease association prediction (EDTMDA) to infer novel miRNA–disease associations which integrated ensemble learning, matrix factorization and dimensionality reduction to obtain final prediction results. Thirdly, the random walk-based methods such as RWRMDA^18^, MIDP&MIDPE^19^, NTSMDA^20^ should be mentioned. Recently, several extended random walk based methods, for examples Le et al.’s^21^ and BRWH^22^, have been developed to address the problem of predicting miRNA–disease associations. Niu et al.^23^ presented a Random Walk and Binary Regression based miRNA–disease association prediction (RWBRMDA) method which extracted features for each miRNA from Random Walk with Restart on the integrated miRNA similarity network for binary logistic regression. Li et al.^24^ used a network projection based dual random walk with restart (NPRWR) model to predict miRNA–disease associations. Nevertheless, the walk probabilities of each linked neighbor node of the disease or miRNA node in line with its degree was identically accredited in most of above random walk-based methods. And almost of the diseases or miRNAs without any known associated miRNAs or diseases could not be effectively predicted.

Although existing computational methods have made immense beneficences to reveal disease‐related miRNAs, but they still contain some limitations which could be improved to achieve more decisive performance. One of these limitations is the problem of sparsity and incompletion of data that affected prediction accuracies. In recent years, a weighted K-nearest known neighbors (WKNKN) algorithm was usually used as a pre-processing step to eliminate unknown values in miRNA–disease association set as in the studies of Ezzat et al.^25^, Gao et al.^26^, Wu et al.^27^, and Li et al.^28^. It relied on the fact the number of known miRNA‐disease associations are very limited in comparison with the number of non-interacting miRNA–disease pairs which are unknown cases that could potentially be accurate associations in the training datasets. In these studies, a new miRNA or disease’s association profile was predicted using its similarities to other miRNAs or diseases, respectively, to reduce unfavorable impact of a large number of missing associations^25,26^.

Recently, Luo J. and Long Y. extended random walk with restart algorithm to explore most potential microbe-disease associations based on a heterogeneous network composed of Gaussian kernel microbe similarity network, Gaussian kernel disease similarity network, and known disease-microbe associations network^29^. This method achieved a desirable performance in predicting microbe-disease associations. However, as mentioned by the authors, its performance could be improved by adding other types of prior biological information such as microbe functional similarity, disease semantic similarity, and disease symptom similarity networks. Additionally, its performance could be superior if the sparsity data problem was solved.

Inspired by the extended random walk with restart algorithm and to promote the performance with the addition of multi-types of biological information and solve the sparsity data problem as indicated in NTSHMDA method^29^, in this paper, we proposed a new method to predict potential miRNA–disease associations using improved random walk with restart and integrating multiple similarities (RWRMMDA). There are three main contributions of our study. First, we integrated multiple similarity networks to build two heterogeneous networks in disease and miRNA spaces, respectively, to designate different walk probabilities to each related neighbor node of the disease or miRNA node in line with its degree in different spaces. Second, we solved the problem of sparsity and incompletion of data to reduce negative impact of a large number of missing associations by using a WKNKN algorithm as a pre-processing step. Finally, we improved the extended random walk with restart algorithm based on miRNA similarity-based and disease similarity-based heterogeneous networks to calculate miRNA–disease association prediction probabilities. The experiments based on the dataset of miRNA–disease associations which was downloaded from the HMDD V2.0 database^30^ containing 5430 experimentally verified associations between 383 diseases and 495 miRNAs as in PMFMDA^4^, miRNA functional similarities and disease semantic similarities showed that our proposed method (RWRMMDA) achieved a decisive performance. In details, RWRMMDA achieved global LOOCV AUC (Area Under Roc Curve) and AUPR (Area Under Precision-Recall Curve) values of 0.9882 and 0.9066 respectively. Additionally, its best AUC and AUPR values, proven by statistical tests, are 0.9855 and 0.8642, respectively, under fivefold-cross-validation experiments. Its performance is superior to other state of the art methods as NTSHMDA^29^, PMFMDA^4^, IMCMDA^13^ and MCLPMDA^14^. It could be considered as a forceful and valuable tool to infer miRNA–disease associations.

## Materials and methods

### Method overview

**Figure 1:**
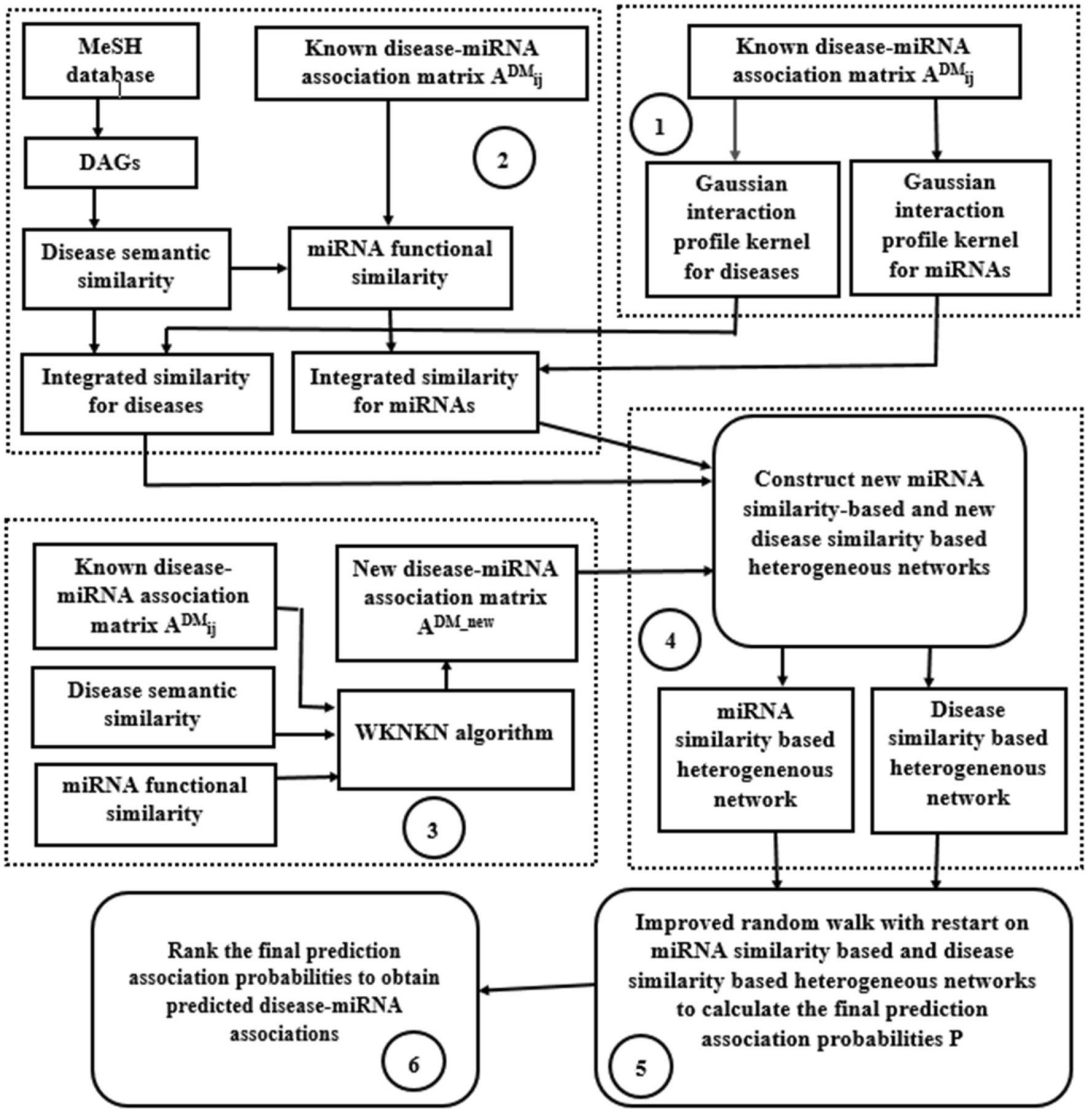
The workflow of the proposed method (RWRMMDA).

In this paper, we proposed a new method to predict potential miRNA–disease associations using improved random walk with restart and integrating multiple similarities (RWRMMDA). The workflow of RWRMMDA is shown in Fig. 1. In overview, RWRMMDA based on the known miRNA–disease associations, miRNA functional similarity and disease semantic similarity information. It contains six stages. At the first stage, we calculated Gaussian Interaction Profile Kernel Similarity for miRNAs and diseases. At second stage, we figured out the Integrated Similarity for miRNAs and diseases. At third stage, we performed a weighted K-nearest known neighbors (WKNKN) algorithm as a preprocessing step to exclude unknown missing values in miRNA–disease association set. In other words, it reduced the impact of sparsity data problem. During the fourth stage, we constructed two miRNA similarity based and disease similarity based heterogeneous networks. Next, we handled an improved random walk with restart algorithm on miRNA similarity-based and disease similarity-based heterogeneous networks to calculate the final prediction probabilities. Finally, we ranked the prediction scores in descending order to obtain the most potential disease associated miRNAs.

### Human miRNA–disease associations

We used an adjacency matrix ${A}^{DM}$ to express the known miRNA–disease associations which were downloaded from the HMDD V2.0 database^30^ and contained 5430 experimentally verified associations between 383 diseases and 495 miRNAs. Especially, if the association between disease ${d}_{i}$ and miRNA ${m}_{j}$ was experimentally verified, we represent the element ${A}_{ij}^{DM}$ to be equal to 1, otherwise ${A}_{ij}^{DM}$ is equal to 0. Hence, a binary vector which indicates the associations between disease ${d}_{i}$ and each miRNA is represented by the *i*th row of ${A}^{DM}$, and a binary vector reflects the associations between miRNA ${m}_{j}$ and each disease is represented by the *j*th column of ${A}^{DM}$_._

### Disease semantic similarity

Disease semantic similarity was estimated according to the literatures^4,17,31^. We gathered the relationships of various diseases based on the hierarchical directed acrylic graphs (DAGs) by downloading MeSH descriptors from the National Library of Medicine (http://www.ncbi.nlm.nih.gov/). DAGs are usually used to measure the similarity among diseases. For instance, for a disease d, its directed acrylic graph is given by $DAG\left(d\right)=(d, {TA}_{d}, {EC}_{d})$, where ${TA}_{d}$ indicates the set of the disease *d*’s ancestors and d itself, and ${EC}_{d}$ symbolizes the set of edges which point to child nodes from parent nodes in the MeSH tree. Therefore, the semantic contribution of disease *t* to disease *d* is as in the following equation

1 $$ D_{d} \left( t \right) = \left\{ {\begin{array}{*{20}l} t \hfill & {if\;t = d} \hfill \\ {\max \left\{ {\Delta *D_{d} \left( {t^{\prime}} \right)| t^{\prime} \in children\, of\, t} \right\}} \hfill & {if\;t \ne d} \hfill \\ \end{array} } \right. $$

where $\Delta$ symbolizes a predefined semantic contribution factor with values range from 0 to 1. According to Wang et al.^31^, Xu et al.^4^ and Chen et al.^17^, in this paper, we set $\Delta$ equal to 0.5. We calculated the semantic similarity between diseases based on the assumption that two diseases having larger parts in their DAGs favor to have higher semantic similarity as in formula (2).

2 $$DSS\left({d}_{i}, {d}_{j}\right)=\frac{{\sum }_{i\in {TA}_{{d}_{i}} \cap {TA}_{{d}_{j}}}({D}_{{d}_{i}}\left(t\right)+{D}_{{d}_{j}}\left(t\right))}{{\sum }_{t \in {TA}_{{d}_{i}}}{D}_{{d}_{i}}\left(t\right)+ {\sum }_{t\in {TA}_{{d}_{j}}}{D}_{{d}_{j}}(t)}$$

### miRNA functional similarity

As previous studies^4,31^, in this paper, the functional similarity measurements were used to represent miRNA functional similarities among miRNAs. Especially, let any two miRNAs ${m}_{i}$ and ${m}_{j}$ associated disease sets be the ${DTT}_{i}=\left\{{d}_{i1},{d}_{i2}, \dots , {d}_{ik}\right\}$ and ${DTT}_{j}=\left\{{d}_{j1},{d}_{j2}, \dots , {d}_{jl}\right\}$, respectively. Similar to Wang et al.^31^ and Xu et al.^4^, we firstly used $SS\left(d,DTT\right)={}_{{d}_{i \in DTT}}{}^{max}DSS(d, {d}_{i})$ to depict the similarity between a disease *d* and *DTT* set. Then, the similarity between ${m}_{i}$ and ${m}_{j}$ was computed as follows:

3 $$MFS\left({m}_{i}, {m}_{j}\right)=\frac{{\sum }_{m=1}^{k}SS\left({d}_{im}, {DTT}_{j}\right)+ {\sum }_{n=1}^{l}SS({d}_{jn}, {DTT}_{i})}{k+l}$$

**Figure 2:**
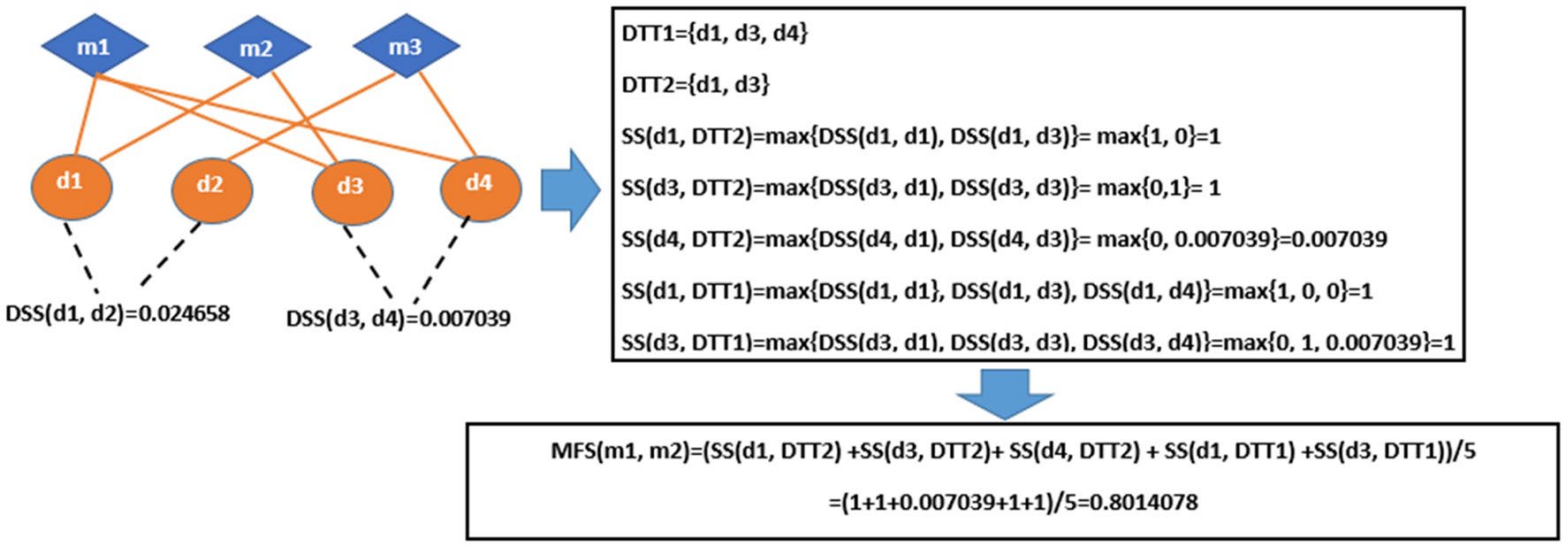
Illustration of calculating miRNA functional similarity.

The illustration of calculating miRNA functional similarity is shown in Fig. 2.

### Gaussian interaction profile kernel similarity for miRNAs and diseases

According to literatures^4,17^, we computed Gaussian interaction profile kernel similarity for miRNAs and diseases relied on the known association adjacency matrix ${A}^{DM}$. Suppose that the vector associated with disease ${d}_{i}$ in ${A}^{DM }$ is represented by ${A}^{DM}\left({d}_{i}\right)$ to reflect the i-th row of ${A}^{DM}$ adjacency matrix. Similarly, the vector associated with miRNA ${m}_{j}$ is repesented by ${A}^{DM}({m}_{j})$ which means the j-th column of ${A}^{DM}$ adjacency matrix. Then, the Gaussian interaction profile kernel similarity between disease ${d}_{i}$ and disease ${d}_{j}$ was computed as follows:

4 $$GIPdisease({d}_{i},{d}_{j})=\text{exp}(-{\gamma }_{d}{\Vert {A}^{DM}\left({d}_{i}\right)-{A}^{DM}\left({d}_{j}\right)\Vert }^{2})$$

where ${\gamma }_{d}$ signifies a kernel bandwidth’s adjustment parameter and it is updated as follows:

5 $${\gamma }_{d}=\frac{{\gamma }_{d}^{{\prime}}}{\frac{1}{{n}_{d}}{\sum }_{i=1}^{{n}_{d}}{\Vert {A}^{DM}\left({d}_{i}\right)\Vert }^{2}}$$

here ${\gamma }_{d}^{{\prime}}$ is widely set to 1 as in previous studies^4,17^.

In a similar way, we calculated the Gaussian interaction profile kernel similarity between miRNA ${m}_{i}$ and miRNA ${m}_{j}$ as follows:

6 $$GIPmiRNA({m}_{i},{m}_{j})=\text{exp}(-{\gamma }_{m}{\Vert {A}^{DM}\left({m}_{i}\right)-{A}^{DM}\left({m}_{j}\right)\Vert }^{2})$$

where ${\gamma }_{m}$ signifies a kernel bandwidth’s adjustment parameter and it is updated as follows:

7 $${\gamma }_{m}=\frac{{\gamma }_{m}^{{\prime}}}{\frac{1}{{n}_{m}}{\sum }_{i=1}^{{n}_{m}}{\Vert {A}^{DM}\left({m}_{i}\right)\Vert }^{2}}$$

here ${\gamma }_{m}^{{\prime}}$ is widely set to 1 as in previous studies^4,17^.

### Integrated similarity for miRNAs and diseases

We could not attain DAGs for all diseases though the disease semantic similarity was determined based on DAGs as mentioned before. Therefore, we could not assess disease semantic similarity in case of the specific disease without DAGs. Consequently, to measure all disease similarity information, we incorporated disease semantic similarity with Gaussian interaction profile kernel according to previous studies^4,32^ as follows:

8 $$ ISD\left( {d_{i} , d_{j} } \right) = \left\{ {\begin{array}{*{20}l} {DSS\left( {d_{i} , d_{j} } \right)} \hfill & {if\;d_{i} \;and\;d_{j} \;has\;semantic\;similarity} \hfill \\ {GIPdisease\left( {d_{i} , d_{j} } \right)} \hfill & {otherwise} \hfill \\ \end{array} } \right. $$

Similarly, integrated miRNA similarity was computed according to previous studies^4,32^ as follows:

9 $$ ISM\left( {m_{i} , m_{j} } \right) = \left\{ {\begin{array}{*{20}l} {MFS\left( {m_{i} , m_{j} } \right)} \hfill & {if\;m_{i} \;and\;m_{j} \;has\;functional\;similarity} \hfill \\ {GIPmiRNA\left( {m_{i} , m_{j} } \right)} \hfill & {otherwise} \hfill \\ \end{array} } \right. $$

### Weighted K-nearest known neighbors algorithm

**Figure 3:**
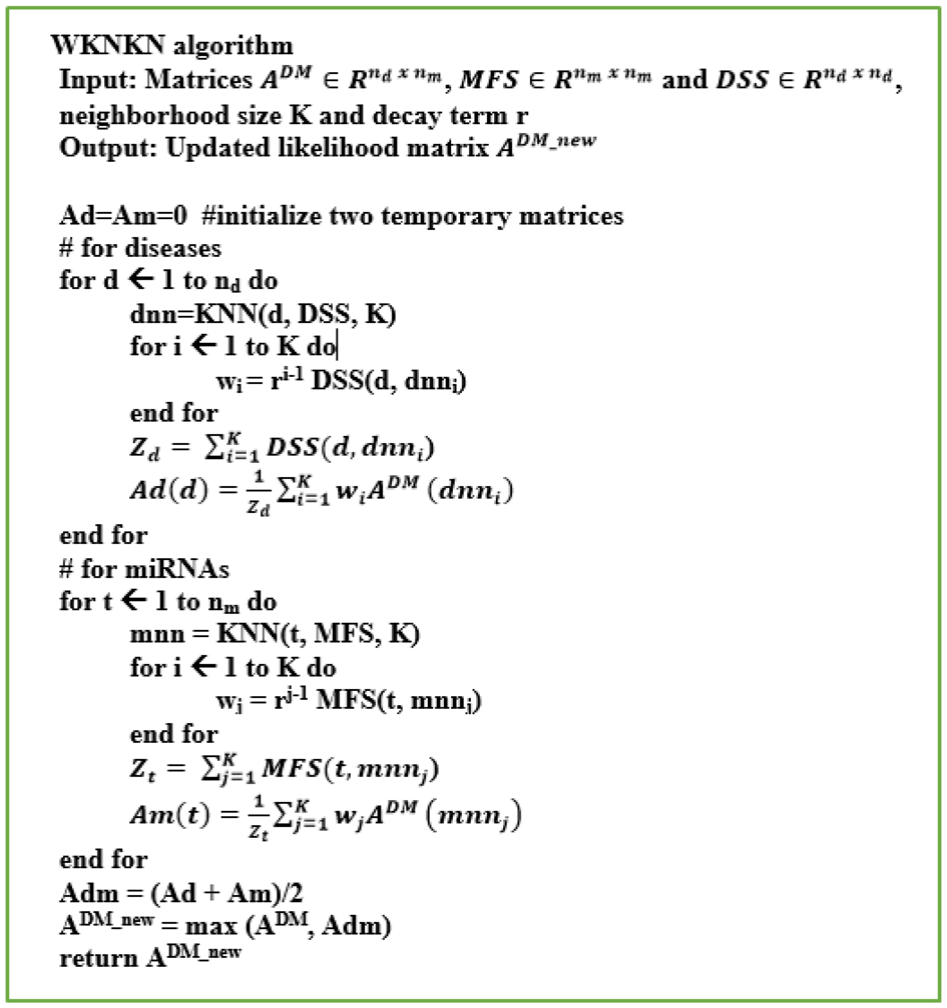
The WKNKN algorithm.

We utilized a WKNKN algorithm introduced in^25,28^ as a pre-processing step to exclude unknown values in miRNA–disease association set. It based on the known neighbors’ information by considering the fact that many of the non-interacting miRNA–disease pairs in ${A}^{DM}$ are unknown cases that could potentially be truthful associations. Particularly, WKNKN replaces ${A}_{ij}^{DM}=0$ with an interaction likelihood continuous value in the range from 0 to 1 as follows. Firstly, for each disease ${d}_{i}$, we selected the semantic similarities with K known diseases which are nearest to ${d}_{i}$ and their corresponding interaction profiles to quantify the interaction likelihood profile for disease ${d}_{i}$. Secondly, for each miRNA ${m}_{j}$, we chose its functional similarities with K known miRNAs which are nearest to ${m}_{j}$ and their corresponding interaction profiles to estimate the interaction likelihood profile for miRNA ${m}_{j}$. And finally, if ${A}_{ij}^{DM}=0$, we changed it by averaging the two interaction likelihood profiles. Figure 3 contains the pseudocode that describes the above steps in detail in which *r* is a decay term where *r* ≤ *1*, and *KNN()* returns the K-nearest known neighbors in descending order based on their similarities to ${d}_{i}$ or ${m}_{j}.$

### Construct miRNA similarity-based and disease similarity based heterogeneous networks

**Figure 4:**
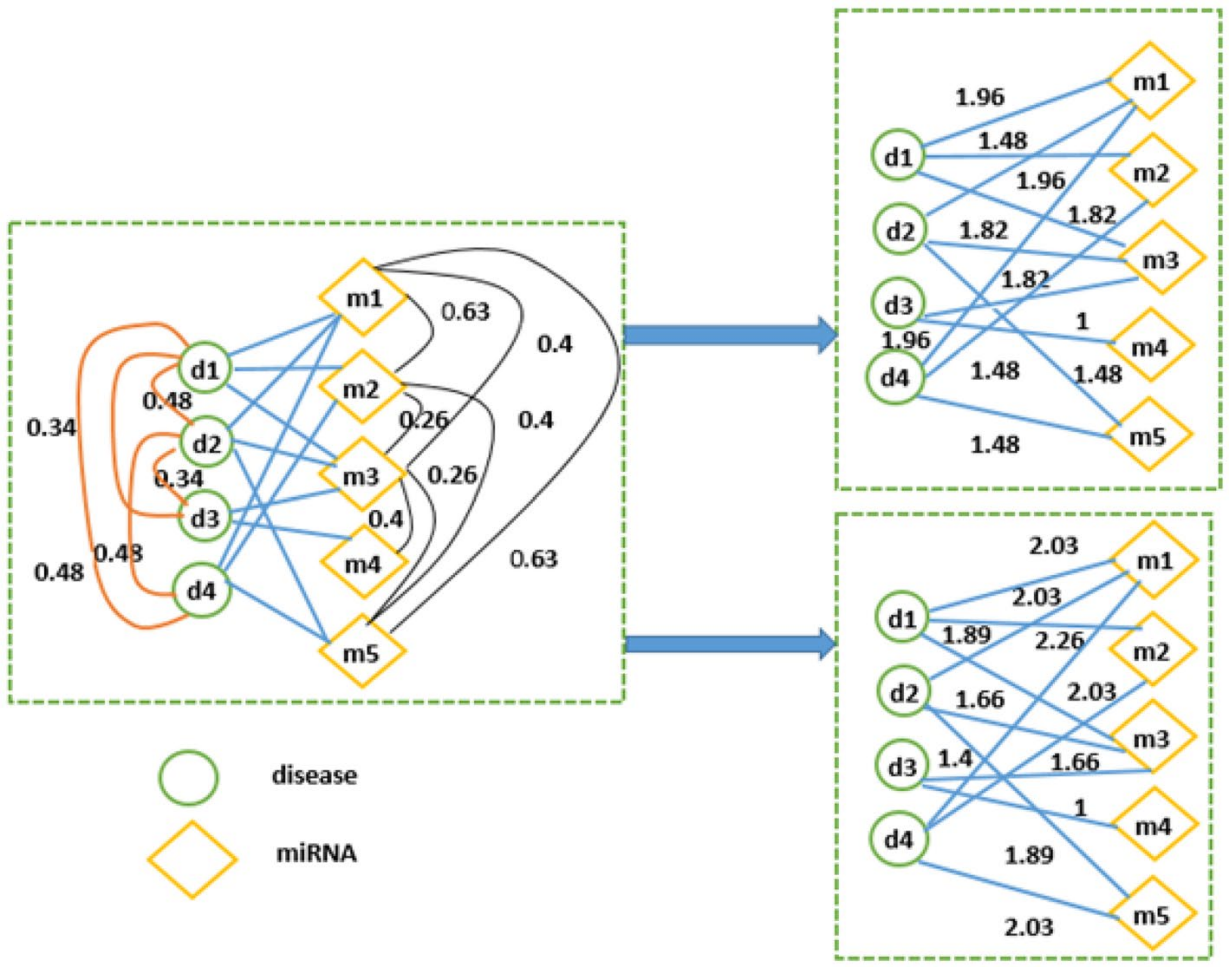
Illustrations of the process of weight assignment in disease space and miRNA space.

Normally, the transition probabilities from a disease (miRNA) node to each related neighbor miRNA (disease) are equally allocated while the total of the probabilities is equal to 1 in the common random walk with restart (RWR) algorithms^18–20^. However, the tends of degree to be related with different miRNAs or diseases corresponding to a given disease or miRNA literally exists difference^29,33^. For instance, a number of associations between a given disease ${d}_{i}$ and many related miRNAs show different similarities among them while remained ${d}_{i}$-associated miRNAs do not have or have sparse similarities to other miRNAs associated with ${d}_{i}$. Therefore, we suppose that a disease or miRNA has stronger relation with miRNA or disease to which a larger number of the remaining miRNAs or diseases are similar among miRNAs or diseases associated with the disease or miRNA^29^. Based on that hypothesis, we incorporated topological similarity with semantic similarity for a disease or with functional similarity for a miRNA to measure the tends of degree to be related of a disease (miRNA) to a miRNA (disease)^29,33^. We determined the edges’ weights in miRNA–disease association network which reflect the related degree of actual association based on integrated similarity for diseases and integrated similarity for miRNAs, respectively as follows. Firstly, a bipartite graph which consists disease nodes and miRNA nodes was constructed. Secondly, when the walker moves from disease network to miRNA network, we selected the possibility of targeted miRNA node ${m}_{j}$ *(j* = *1, 2, …, n*_*m*_*)* for a specific disease node ${d}_{i}$ (*i* = *1, 2, …, n*_*d*_) totally depends on the similarities between ${m}_{j}$ and all neighbor ${d}_{i}$-related miRNA nodes including ${m}_{j}$ ^29^. Analogously, for a specific miRNA node ${m}_{j}$ *(j* = *1, 2, …, n*_*m*_*),* when the walker moves to disease network from miRNA network, we selected the possibility of targeted disease node ${d}_{i}$ *(i* = *1, 2,…, n*_*d*_*)* totally bases on the similarities between ${d}_{i}$ and all neighbor ${m}_{j}$_-_related disease nodes including ${d}_{i}$ ^29^. Figure 4 illustrates a simple example of the process of weight assignment in disease and miRNA spaces, respectively. Finally, we redefined two new integrated adjacency matrices ${A}^{\text{DMdiseasebase}}$ and ${A}^{\text{DMmirnabase}}$ based on the integrated similarity *ISD* matrix for diseases, integrated similarity *ISM* matrix for miRNAs and ${A}^{DM\_new}$ adjacency matrix as in the following equations:

10 $${A}^{\text{DMdiseasebase}}(i,j)= \sum_{k=1}^{{n}_{d}}IDS(i,k){A}^{\text{DM}\_\text{new }}(k,j)$$

11 $${A}^{\text{DMmirnabase}}(i,j)= \sum_{k=1}^{{n}_{m}}{A}^{\text{DM}\_\text{new }}(i,k)IMS(k,j)$$

### Improved random walk with restart to predict miRNA–disease associations

Firstly, we defined a transition probability matrix from disease network to miRNA network ${T}_{DM}$ and a transition probability matrix from miRNA network to disease network ${T}_{MD}$ based on the two new integrated adjacency matrices identified previously as follows:

12 $${T}_{DM}(i,j)= \varphi \frac{{A}^{{\text{DM}}_{\text{new}}}\left(\text{i},\text{j}\right)* {A}^{\text{DMmirnabase}}(i,j)}{{\sum }_{l=1}^{{n}_{m}}{A}^{{\text{DM}}_{\text{new}}}\left(\text{i},\text{l}\right)*{A}^{\text{DMmirnabase}}(i,l)}$$

13 $${T}_{MD}\left(i,j\right)= \varphi \frac{{A}^{{\text{DM}}_{\text{new}}}\left(\text{i},\text{j}\right)* {A}^{\text{DMdiseasebase}}(i,j)}{{\sum }_{l=1}^{{n}_{d}}{A}^{{\text{DM}}_{\text{new}}}\left(l,\text{j}\right)*{A}^{\text{DMdiseasebase}}(l,j)}$$

where $\varphi \in \left(\text{0,1}\right)$ is the jumping probability of random walker among these two different networks^29^.

Secondly, we defined a disease transition probability matrix ${W}_{d}$ to represent the transition probabilities from a disease node to all neighbor disease nodes in disease network in which the element ${W}_{d}\left(i,j\right)$ signifies the jumping probability from disease ${d}_{i}$ to disease ${d}_{j}$ as in Eq. (14).

14 $$ W_{d} \left( {i,j} \right) = \left\{ {\begin{array}{*{20}l} {\left( {1 - \varphi } \right)\frac{{IDS\left( {i,j} \right)}}{{\mathop \sum \nolimits_{k = 1}^{{n_{d} }} IDS\left( {i,k} \right) }}} \hfill & {if\;\sum\nolimits_{t = 1}^{{n_{m} }} {A^{{{\text{DM}}_{{{\text{new}}}} }} \left( {{\text{i}},{\text{t}}} \right) \ne 0} } \hfill \\ {\frac{{IDS\left( {i,j} \right)}}{{ \mathop \sum \nolimits_{k = 1}^{{n_{d} }} IDS\left( {i,k} \right) }}} \hfill & {otherwise} \hfill \\ \end{array} } \right. $$

Furthermore, the miRNA network transition probability matrix ${W}_{m}$ can be constructed as follows:

15 $$ W_{m} \left( {i,j} \right) = \left\{ {\begin{array}{*{20}l} {\left( {1 - \varphi } \right)\frac{{IMS\left( {i,j} \right)}}{{\sum\nolimits_{k = 1}^{{n_{m} }} {IMS\left( {i,k} \right)} }}} \hfill & {if\;\sum\nolimits_{t = 1}^{{n_{d} }} {A^{{{\text{DM}}_{{{\text{new}}}} }} \left( {t,{\text{i}}} \right) \ne 0} } \hfill \\ {\frac{{IMS\left( {i,j} \right)}}{{ \mathop \sum \nolimits_{k = 1}^{{n_{m} }} IDS\left( {i,k} \right)}}} \hfill & {otherwise} \hfill \\ \end{array} } \right. $$

Thirdly, instead of using the vector form of initial probability as in common RWR algorithms^18–20^, and inspired by the extended RWR proposed by Luo and Long^29^, we defined the initial probability matrix

16 $${P}_{0}= \left[\begin{array}{cc}(1-\delta )P{D}_{0}& 0\\ 0& \delta {PM}_{0}\end{array}\right]$$

of heterogenous network to perform improved random walk with restart with supposition that all miRNA–disease associations could be concurrently produced, where ${PD}_{0}$ and ${PM}_{0}$ are the diagonal matrices with ${PD}_{0}\left(i, i\right)=1/{n}_{d}$ and ${PM}_{0}\left(j, j\right)=1/{n}_{m}$ serve as the normalized probabilities of disease and miRNA seed nodes and $\delta$ is the weight factor used to point out the importance level or impact factor of two sub-networks which are represented by ${A}^{\text{DMdiseasebase}}$ and ${A}^{\text{DMmirnabase}}$ matrices.

And then, we defined a new transition probability matrix ${W}_{\text{newTP}\_\text{DM}}$ of heterogeneous network relied on disease similarity-based network as follows:

17 $$ W_{newTP\_DM} = \left[ {\begin{array}{*{20}l} {W_{d} } \hfill & {T_{DM} } \hfill \\ {T_{{DM^{\prime } }} } \hfill & {W_{m} } \hfill \\ \end{array} } \right] $$

and a new transition probability matrix ${W}_{\text{newTP}\_\text{MD}}$ of heterogeneous network depended on miRNA similarity-based network as follows:

18 $$ W_{newTP\_MD} = \left[ {\begin{array}{*{20}c} {W_{d} } & {T_{{MD^{\prime } }} } \\ {T_{MD} } & {W_{m} } \\ \end{array} } \right] $$

where ${T}_{DM}$, and ${T}_{MD}$, are the transpose matrices of ${T}_{DM}$ and ${T}_{MD}$ respectively. From the new transition probability matrices and initial transition probability matrix, the improved random walk with restart can be identified as follows:

19 $${P1}_{t+1}=\left(1-\gamma \right){W}_{newTP\_DM}{P1}_{t}+ \gamma {P}_{0}$$

20 $${P2}_{t+1}=\left(1-\gamma \right){W}_{newTP\_MD}{P2}_{t}+ \gamma {P}_{0}$$

where ${P1}_{t}$ and ${P2}_{t}$ illustrate prediction matrices which reflect the probability values of all miRNA–disease associations at the *t* time step, and $\gamma$ stands for the restart probability, $\gamma \in \left({0,1}\right).$ We again and again executed the improved random walk process on the heterogeneous network until convergence, generally, the *t* time is set to 10 as in^29^.

Finally, the final prediction matrix *P* is defined as:

21 $$P=\left(1-\delta \right)*P1+ \delta *P2$$

in which the elements of *P* reveal the score of associations between disease nodes and miRNA nodes would be produced simultaneously.

### Rank the final prediction score of associations to obtain predicted miRNA–disease associations

For a given disease, we ranked all candidate miRNAs’ score of associations in descending order to obtain the most possible miRNA–disease associations. The candidate with higher score will have more chance to be verified in the future.

### Ethics approval and consent to participate

Not applicable. The study does not involve human subjects, only used public data.

## Results

### Performance measures

We appraise our method’s performance in inferring miRNA–disease associations by doing the fivefold cross-validation experiments and global LOOCV and measure the Area under roc curve (AUC)^34^ and the Area under precision-recall curve (AUPR)^35^ as described in the followings.

To measure AUC values, we computed the false positive rate (FPR) and true positive rate (TPR) values where FPR is used to indicate the proportion of the real negative samples in predicted positive samples to all negative samples. And, TPR signifies the proportion of the real positive samples in all predicted positive samples. The FPR and TPR are gauged by the following equations:

22 $$FPR=\frac{FP}{FP+TN}$$

23 $$TPR= \frac{TP}{TP+FN}$$

where TP (true positive) specifies that a positive sample is precisely forecasted as positive sample; FN (false negative) depicts that a positive sample is falsely predicted as negative sample; FP (false positive) symbolizes that a negative sample wrongly predicted as positive sample; TN (true negative) shows that a negative sample is perfectly concluded as negative sample. We used TPR as vertical axis and FPR as horizontal axis to figure the receiver operating characteristic (ROC) curve^34^.

As mentioned by Takaya Saito and Marc Rehmsmeier^35^, in case of Evaluating Binary Classifiers on Imbalanced Datasets, the Precision-Recall is more informative than the ROC. Therefore, we also draw Precision-Recall curve and calculate the AUPR value to evaluate prediction performance. The Precision depicts the percentage of the accurately predicted positive samples in all predicted positive samples whereas the Recall reflects the percentage of the accurately predicted positive samples in all real positive samples. Precision and Recall are computed as follows:

24 $$Precision=\frac{TP}{TP+FP}$$

25 $$Recall=\frac{TP}{TP+FN}$$

### Evaluating the AUC and AUPR under fivefold cross validation

**Figure 5:**
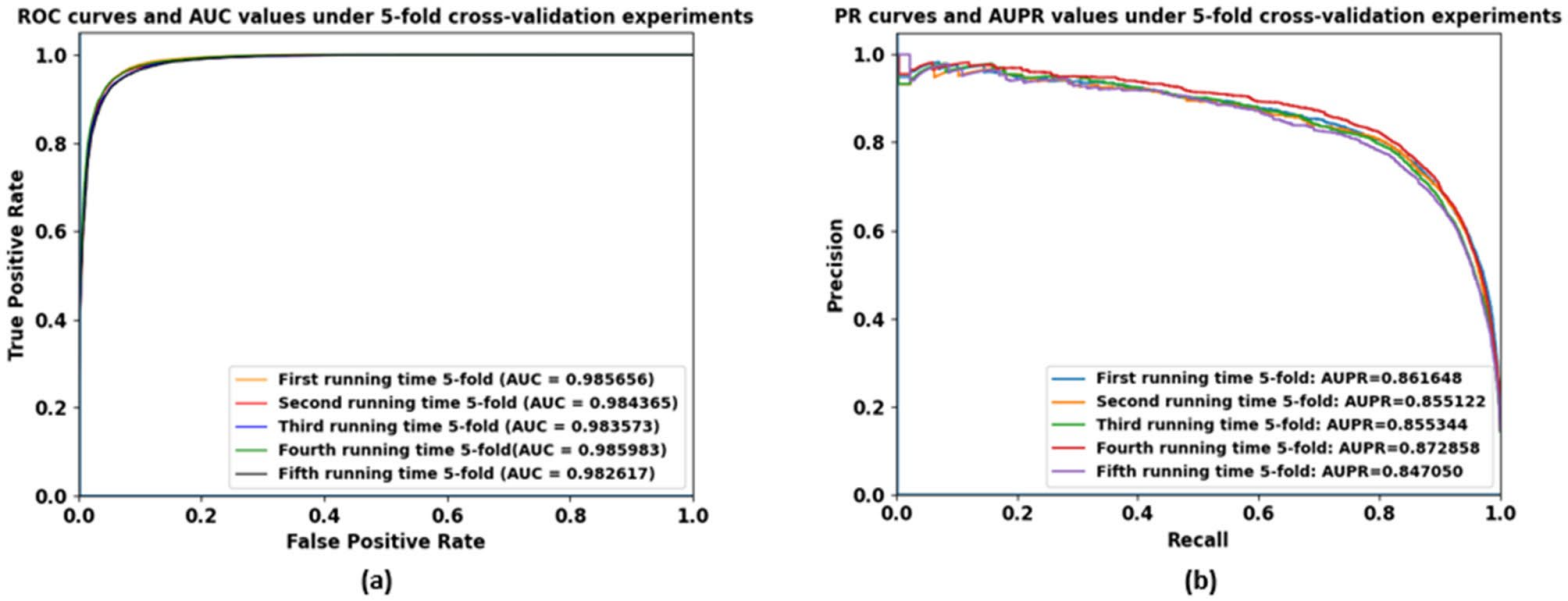
ROC curves and AUC values (**a**) and PR curves and AUPR values (**b**) in 5 running times of fivefold cross-validation experiments.

In fivefold cross-validation experiments, firstly we considered the known miRNA–disease associations as positive samples and the remained unknown associations as negative samples. Secondly, we randomly partitioned all positive and negative samples in known adjacency matrix ${A}^{DM}$ into five equal parts to perform fivefold cross-validation. Thirdly, in each experimental running time, we took four parts of positive and negative samples for training and the last part for testing. The elements’ values which are equal to 1 in the part used for testing were changed to 0. Fourthly, we recalculated *Final_score* in each running time. Finally, we matched the *Final_score* in each running time with the new adjacency matrix attained by applying WKNKN algorithm to figure out AUC and AUPR values. To increase the reliability of AUC and AUPR values, we again and again performed fivefold cross-validation experiments for 25 times and computed AUC and AUPR values to obtain final results. Our proposed model achieved best AUC value of 0.9855 and obtained the best AUPR value of 0.8642 after 25 times under fivefold cross-validation experiments. These values are proven by statistical tests. We already performed One sample T Test with N = 25 at confidence level of 95%. The details results of statistical tests on One sample T Test of AUC and AUPR are shown in Table 1. Figure 5 illustrates ROC curves and AUC values (a) and PR curves and AUPR values (b) in five running times of fivefold cross-validation experiments.

**Table 1:** AUC and AUPR one-sample T test.

	N	Mean	Std. deviation	Std. Error Mean	AUC test value = 0.9855 AUPR test value = 0.8642					
					t	df	Sig. (2-tailed)/p-value	Mean difference	95% confidence interval of the difference	
									Lower	Upper
AUC	25	0.984908	0.0011909	0.0002382	− 2.485	24	0.020	− 0.0005920	− 0.001084	− 0.000100
AUPR	25	0.8595	0.017862	0.002572	− 2.181	24	0.039	− 0.0047040	− 0.009156	− 0.000252

### Evaluating AUC and AUPR under global LOOCV experiments

**Figure 6:**
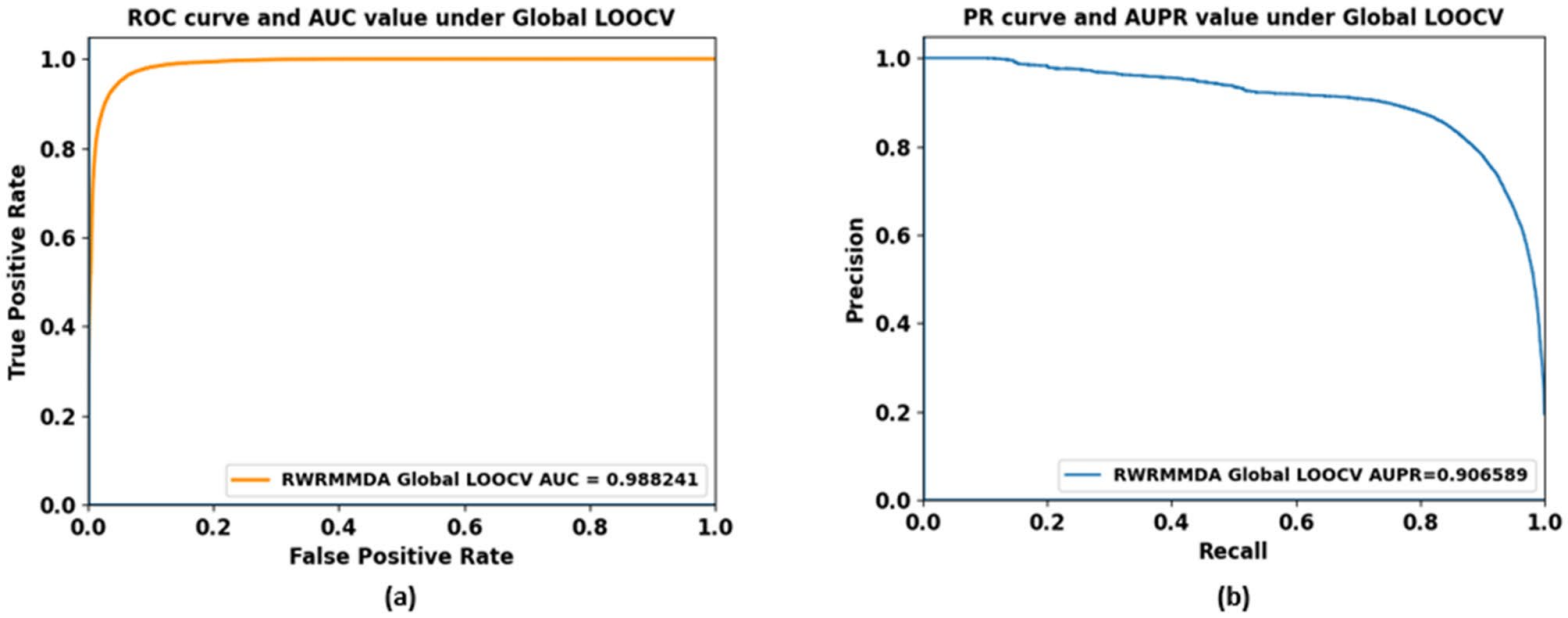
ROC curve and AUC value (**a**) and PR curve and AUPR value (**b**) under global LOOCV experiment.

Leave-one-out cross validation (LOOCV) was normally used to evaluate global prediction ability of a model^4,36^. In this paper, we performed global LOOCV experiments by removing each known miRNA–disease association in turn as a testing sample and all remaining associations as training samples. Then we recalculated the final prediction matrix P in each running time to evaluate prediction performance. The global LOOCV prediction performance of our proposed method achieved AUC value of 0.9882 and AUPR value of 0.9066 as demonstrated in Fig. 6. They are slight higher than AUC and AUPR values under fivefold cross validation because the number of known associations which were removed in each experimental running time of fivefold cross validation is bigger than in global LOOCV experiment.

### Effects of parameters

The proposed model contains five parameters which effect on the performance of the model. In other words, the best results with above AUC and AUPR values could be obtained by modifying the union of multiple parameters with their different values.

#### Two parameters from WKNKN

Considering that there are some unknown miRNA–disease associations in the matrix A^DM^_ij_, the WKNKN algorithm was used as a pre-processing step to exclude unknown values in miRNA–disease association set based on their known neighbors. The K parameter reflects the number of nearest known neighbors, r means a decay term where r ≤ 1. In this study, we mainly focus on the influence of number of nearest known neighbors to reduce the impact of sparsity data problem. The more nearest known neighbors were chosen, the more associations between diseases and miRNAs would be added into the heterogeneous network. And the impact of sparsity data problem would be reduced. However, when the number of added associations was too big, the imbalanced data problem would again appear. Therefore, the two parameters would be determined to the optimal value before performing improved random walk on heterogeneous networks. In our experiments, we again and again changed the value of K and r to choose the optimal values. And it showed that AUC and AUPR achieve the best values when K = 5 and r = 0.7. It is similar to the result in NPCMF method^26^. Table 2 shows the evaluation index changes when K was fixed to 5 and r ranged from 0.1 to 0.9 and r was fixed to 0.7 and K range from 1 to 9 when evaluating prediction performance over all samples.

**Table 2:** Evaluation of index changes in WKNKN algorithm.

Index changes	K = 5		Index changes	r = 0.7	
	AUC	AUPR		AUC	AUPR
r = 0.1	0.9528	0.8049	K = 1	0.9503	0.7564
r = 0.2	0.9621	0.8245	K = 2	0.9628	0.8396
r = 0.3	0.9701	0.8434	K = 3	0.9698	0.8431
r = 0.4	0.9767	0.8622	K = 4	0.9761	0.8962
r = 0.5	0.9818	0.8795	K = 5	0.9883	0.9073
r = 0.6	0.9855	0.8946	K = 6	0.987	0.9046
r = 0.7	0.9883	0.9073	K = 7	0.9855	0.9027
r = 0.8	0.9876	0.9058	K = 8	0.9828	0.8979
r = 0.9	0.9875	0.9054	K = 9	0.9798	0.8955

#### Three parameters from improved random walk with restart

When performing improved random walk with restart on heterogeneous networks, there are three parameters which can imply the result performance. The $\varphi$ parameter, $\varphi \in \left({0,1}\right),$ is used to indicate the jumping probability of random walker among two different networks. $\text{The} \delta$ $\text{parameter}$, $\delta \in \left({0,1}\right),$ signifies the weight factor used to present the importance level or impact factor of two sub-networks. The $\gamma$ parameter, $\gamma \in \left({0,1}\right)$, stands for the restart probability. We examined the influences of the three parameters by adjusting them over repeated experiments and then select $\varphi =0.9$, $\delta =0.7 \text{and} \gamma =0.7$ as the optimal combination values in our proposed method.

### Performance comparison with other related models

**Figure 7:**
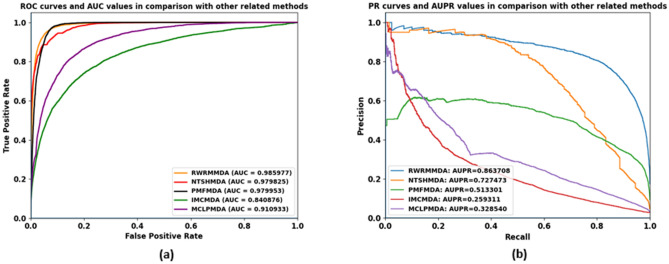
ROC curves and AUC values (**a**) and precision-recall curves and AUPR values (**b**) in comparison with other related approaches.

In comparison with other related approaches to demonstrate the outperformance of our model, we compare our model performance with the performances of NTSHMDA^29^, PMFMDA^4^, IMCMDA^13^ and MCLPMDA^14^ models under best averaged fivefold cross validation experiments The NTSHMDA method contained an extended Random Walk with Restart algorithm which we used in our method. PMFMDA, ICMMDA and MCLPMDA methods used the same miRNA–disease association dataset as in our experiments. The performances of these methods in terms of AUCs and AUPRs are shown in Fig. 7. As can be seen, our proposed approach is superior to all NTSHMDA, PMFMDA, IMCMDA and MCLPMDA methods in AUC measurement of 0.61%, 0.6%, 14.5% and 7.5%, respectively. It is superior to all NTSHMDA, PMFMDA, IMCMDA and MCLPMDA methods in AUPR measurement of 13.62%, 35.04%, 60.44% and 53.52%, respectively. The differences in accuracy values between different methods indicated that our proposed method outperforms all other previous related methods. Especially, in the kind of imbalanced datasets, the significant improvement in AUPR performance prediction showed that our proposed method could be considered to be more informative and reliable than other previous related methods.

**Figure 8:**
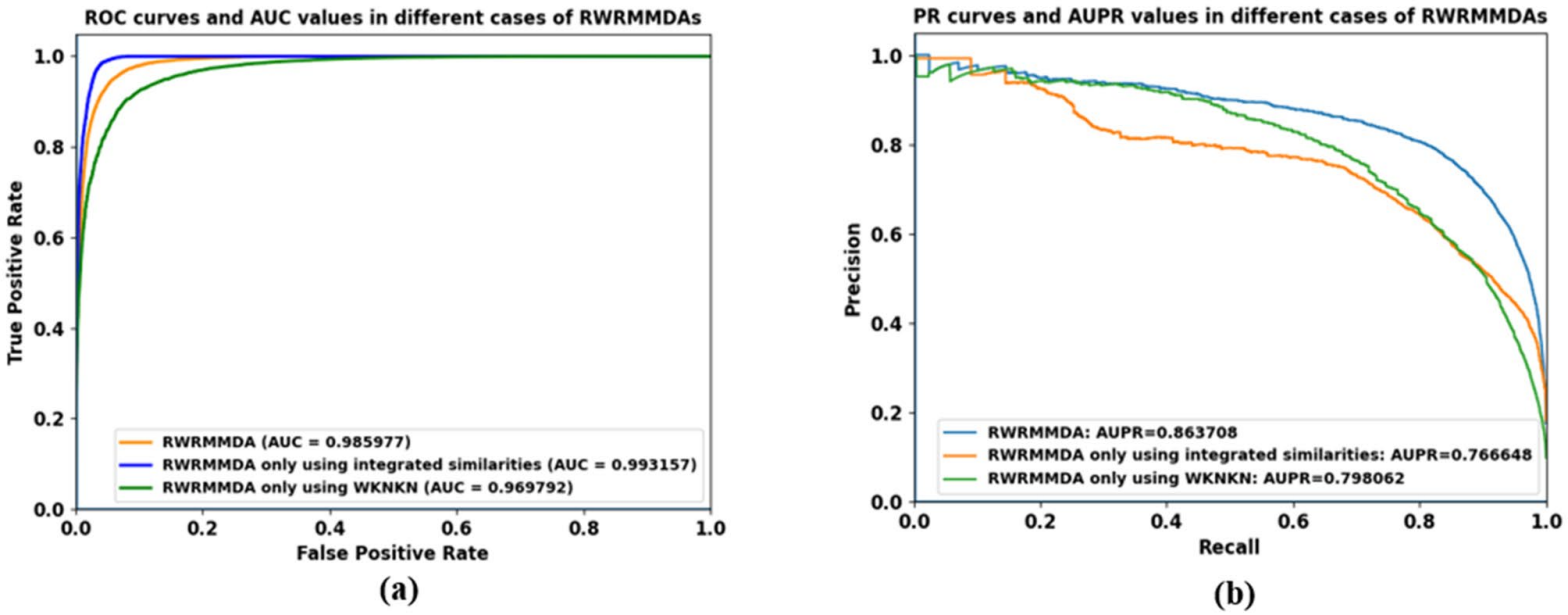
ROC curves and AUC values (**a**) and precision-recall curves and AUPR values (**b**) in different cases of RWRMMDAs.

Additionally, to understand the effects of using WKNKN and integrating multiple similarities independently, we also draw ROC curves and Precision and Recall curves of performing random walk with restart in the cases of (1) using WKNKN as a pre-processing step and not using integrated similarities, and (2) using integrated similarities and not using WKNKN as a pre-processing step. As shown in Fig. 8a, the AUC value of the proposed method seems to be the average of the AUC values of the above cases (1) and (2). And, as illustrated in Fig. 8b, the AUPR value of the proposed method is the highest one in comparison with the above cases. It means that both cases of using WKNKN algorithm as a pre-processing step and using integrated similarities respectively, can increase the AUPR values while using WKNKN algorithm as a pre-processing step can reduce the impact of sparsity data problem when evaluating AUC values.

### Case studies

In addition to fivefold-cross-validation experiments, we also employed some case studies on our proposed approach by doing experiments on all known samples of miRNA–disease associations and for a given disease, the candidate associated miRNAs’ scores are sorted in descending order to have predicted associations. In more details, the case studies on Breast Neoplasms, Carcinoma Hepatocellular and Stomach Neoplasms are constructed to show the ability of our approach in order to infer miRNA–disease associations.

#### Breast neoplasms

Breast Neoplasms is also known as Breast Cancer, it is the leading cause of cancer death in women worldwide. MicroRNAs (miRNAs) have been found to play an important role in breast cancer^37,38^. For example, miR-34 family members in regulating of proliferation, apoptosis, invasion, and metastasis of breast cancer cells^39^. miR-34a inhibits proliferation and migration of breast cancer through down-regulation of Bcl-2 and SIRT1^40^. In this paper, we selected Breast Neoplasms as a case study to demonstrate the ability of our method in inferring miRNA–disease associations. As can be seen in Table 3, in top 40 predicted Breast Neoplasms-associated miRNAs, there is one new miRNA–disease association. This new association has been verified in dbDEMC V2.0 database.

**Table 3:** Top 40 predicted breast neoplasms-associated miRNAs.

Rank	miRNA	Known before	Evidence(s)	Rank	miRNA	Known before	Evidence(s)
1	hsa-mir-298	1	Known association	21	hsa-mir-874	1	Known association
2	hsa-mir-1245a	1	Known association	22	hsa-mir-632	1	Known association
3	hsa-mir-1245b	1	Known association	23	hsa-mir-301b	1	Known association
4	hsa-mir-1323	1	Known association	24	hsa-mir-452	1	Known association
5	hsa-mir-1469	1	Known association	25	hsa-mir-922	1	Known association
6	hsa-mir-181	1	Known association	26	hsa-mir-519d	1	Known association
7	hsa-mir-2355	1	Known association	27	hsa-mir-215	1	Known association
8	hsa-mir-3130	1	Known association	28	hsa-mir-147a	1	Known association
9	hsa-mir-3186	1	Known association	29	hsa-mir-320e	1	Known association
10	hsa-mir-4257	1	Known association	30	hsa-mir-450a	1	Known association
11	hsa-mir-4306	1	Known association	31	hsa-mir-450b	1	Known association
12	hsa-mir-718	1	Known association	32	hsa-mir-320d	1	Known association
13	hsa-mir-505	1	Known association	33	hsa-mir-202	1	Known association
14	hsa-mir-200	1	Known association	34	hsa-mir-345	1	Known association
15	hsa-mir-1915	1	Known association	35	hsa-mir-520b	1	Known association
16	hsa-mir-1471	1	Known association	36	hsa-mir-193a	1	Known association
17	hsa-mir-1258	1	Known association	37	hsa-mir-608	1	Known association
18	hsa-mir-520h	1	Known association	38	hsa-mir-382	0	dbDEMC V2.0
19	hsa-mir-103b	1	Known association	39	hsa-mir-324	1	Known association
20	hsa-mir-299	1	Known association	40	hsa-mir-151a	1	Known association

#### Hepatocellular carcinoma

Hepatocellular carcinoma (HCC) is the most common primary liver malignancy and it is a leading cause of cancer-related death in global^41^. In the United States, HCC is the ninth leading cause of cancer deaths^42,43^. MiRNAs are essential participants and regulators and they also play important roles in the development and progression in HCC^41^. For instances, microRNA-146a inhibits cancer metastasis by downregulating VEGF through dual pathways in hepatocellular carcinoma^44^. miRNA-21 contributes to tumor progression by converting hepatocyte stellate cells to cancer-associated fibroblasts in HCC^45^. By selecting HCC as a case study to illustrate the ability of our approach, it discovered 12 new associations out of top 40 predicted Hepatocellular Carcinoma-associated miRNAs as can be seen in Table 4. To increase the reliability of predicted results, we already checked the evidences of these new predicted associations in dbDEMC V2.0, mirCancer, mirdb (http://mirdb.org/) databases as well as in other literatures. For examples, the new predicted association between hsa-mir-452 miRNA and Hepatocellular carcinoma disease has been verified in dbDEMC V2.0 database and some other published papers^46–48^. For the new predicted association between has-mir-454 and Hepatocellular carcinoma disease, Yu et al.^49^ proved that miR-454 functions as an oncogene by inhibiting CHD5 in hepatocellular carcinoma. Wu et al.^50^ indicated that MicroRNA-655-3p functions as a tumor suppressor by regulating ADAM10 and *β*-catenin pathway in Hepatocellular Carcinoma.

**Table 4:** Top 40 predicted hepatocellular carcinoma-associated miRNAs.

Rank	miRNA	Known before	Evidence(s)	Rank	miRNA	Known before	Evidence(s)
1	hsa-mir-151a	1	Known association	21	hsa-mir-320b	1	Known association
2	hsa-mir-320c	1	Known association	22	hsa-mir-320d	1	Known association
3	hsa-mir-345	1	Known association	23	hsa-mir-320e	1	Known association
4	hsa-mir-452	0	dbDEMC V2.0	24	hsa-mir-365a	1	Known association
5	hsa-mir-454	0	dbDEMC V2.0	25	hsa-mir-365b	1	Known association
6	hsa-mir-655	0	mirCancer	26	hsa-mir-425	1	Known association
7	hsa-mir-484	1	Known association	27	hsa-mir-450a	1	Known association
8	hsa-mir-483	1	Known association	28	hsa-mir-450b	1	Known association
9	hsa-mir-376a	1	Known association	29	hsa-mir-493	1	Known association
10	hsa-mir-144	1	Known association	30	hsa-mir-519d	1	Known association
11	hsa-mir-590	1	Known association	31	hsa-mir-520b	1	Known association
12	hsa-mir-509	0	dbDEMC V2.0	32	hsa-mir-608	1	Known association
13	hsa-mir-765	1	Known association	33	hsa-mir-638	0	dbDEMC V2.0
14	hsa-mir-346	1	Known association	34	hsa-mir-378b	0	http://mirdb.org/
15	hsa-mir-193a	1	Known association	35	hsa-mir-378c	0	dbDEMC V2.0
16	hsa-mir-550a	1	Known association	36	hsa-mir-378d	0	dbDEMC V2.0
17	hsa-mir-105	1	Known association	37	hsa-mir-378e	0	http://mirdb.org/
18	hsa-mir-1290	1	Known association	38	hsa-mir-378f	0	http://mirdb.org/
19	hsa-mir-147a	1	Known association	39	hsa-mir-378g	0	http://mirdb.org/
20	hsa-mir-202	1	Known association	40	hsa-mir-378h	0	http://mirdb.org/

#### Stomach neoplasms

Stomach Neoplasms is also known as Stomach Cancer or Gastric Cancer. It is one of the most common malignant neoplasms worldwide. It has a high incidence and mortality^51^. It is needed to identify sufficiently sensitive biomarkers for Gastric Cancer. MicroRNAs (miRNAs) could be promising potential biomarkers for Gastric Cancer diagnosis. Various studies have indicated important role of the microRNAs in gastric cancers^52,53^. Instantly, microRNA-181a Functions as an Oncogene in Gastric Cancer by Targeting Caprin-1^54^. The development of gastric cancer is affected by MicroRNA-183’s regulating autophagy via MALAT1-miR-183-SIRT1 axis and PI3K/AKT/mTOR signals^55^. With case study of Stomach Neoplasms, our method uncovers 7 new predicted miRNA–disease associations out of top 40 predicted Stomach Neoplasms-associated miRNAs as be shown in Table 5. All of these new predicted miRNA–disease associations have been verified in other databases such as mirCancer, mirDB, dbDEMC V2.0 and other literatures. For examples, Wang et al.^56^ showed that Hsa-mir-152 expression was significantly down regulated in Gastric Cancer cell lines. MicroRNA-338 inhibits growth, invasion and metastasis of Gastric Cancer by Targeting NRP1 Expression^57^.

**Table 5:** Top 40 predicted stomach neoplasms-associated miRNAs.

Rank	miRNA	Known before	Evidence(s)	Rank	miRNA	Known before	Evidence(s)
1	hsa-mir-103a	1	Known association	21	hsa-mir-374a	1	Known association
2	hsa-mir-152	0	dbDEMC V2.0	22	hsa-mir-409	1	Known association
3	hsa-mir-449a	1	Known association	23	hsa-mir-423	0	http://mirdb.org/
4	hsa-mir-338	0	mirCancer	24	hsa-mir-495	1	Known association
5	hsa-mir-374b	1	Known association	25	hsa-mir-513a	1	Known association
6	hsa-mir-421	1	Known association	26	hsa-mir-515	1	Known association
7	hsa-mir-433	1	Known association	27	hsa-mir-516b	1	Known association
8	hsa-mir-519a	1	Known association	28	hsa-mir-519c	1	Known association
9	hsa-mir-650	1	Known association	29	hsa-mir-519e	1	Known association
10	hsa-mir-744	1	Known association	30	hsa-mir-520a	1	Known association
11	hsa-mir-301b	0	dbDEMC V2.0	31	hsa-mir-526a	1	Known association
12	hsa-mir-107	1	Known association	32	hsa-mir-625	1	Known association
13	hsa-mir-128	1	Known association	33	hsa-mir-661	1	Known association
14	hsa-mir-497	1	Known association	34	hsa-mir-302e	1	Known association
15	hsa-mir-296	1	Known association	35	hsa-mir-302f	1	Known association
16	hsa-mir-328	1	Known association	36	hsa-mir-130b	1	Known association
17	hsa-mir-520d	1	Known association	37	hsa-mir-217	0	dbDEMC V2.0
18	hsa-mir-135b	1	Known association	38	hsa-mir-371	0	mirCancer
19	hsa-mir-151b	1	Known association	39	hsa-mir-98	0	dbDEMC V2.0
20	hsa-mir-340	1	Known association	40	hsa-mir-186	1	Known association

#### Predicting new disease-related miRNAs

The dataset used in this study does not contain any new disease or new miRNA. It means that a disease or a miRNA in this dataset has at least one known association with other miRNAs or diseases. Therefore, to demonstrate the proposed method’s performance in predicting new disease-related miRNAs, we conducted two simulated experiments on Lung Neoplasms and Ovarian Neoplasms diseases.

The first simulated experiment was conducted based on Lung Neoplasms. It is also known as Lung Cancer and is the leading cause of cancer deaths worldwide^58^. The clinical applications of miRNAs in lung cancer diagnosis and prognosis have been indicated in many studies^58,59^. In this study, the dataset contained 132 associations between Lung neoplasms and miRNAs. We already removed all known associations related to Lung neoplasms to perform the simulated experiment of predicting new disease-related miRNAs. After performing simulated experiments, we selected top ten predicted miRNAs for Lung cancer to report the performance of our method. As can be seen in Table 6, in top ten predicted miRNAs, our method successfully predicted four known associations and it inferred six new associations. All of six new predicted associations have been confirmed in other databases or literature.

**Table 6:** Top 10 predicted lung neoplasms-associated miRNAs in the simulated experiment for predicting new disease-related miRNAs.

Rank	miRNA	Known before	Evidence(s)	Rank	miRNA	Known before	Evidence(s)
1	hsa-mir-1297	1	Known association	6	hsa-mir-1301	0	dbDEMC V2.0
2	hsa-mir-511	1	Known association	7	hsa-mir-92a	1	Known association
3	hsa-mir-1202	0	dbDEMC V2.0	8	hsa-mir-26	0	PMID: 30687089
4	hsa-mir-1231	0	dbDEMC V2.0	9	hsa-mir-500b	0	dbDEMC V2.0
5	hsa-mir-224	1	Known association	10	hsa-mir-517c	0	dbDEMC V2.0

The second simulated experiment was performed on Ovarian Neoplasms. It is also known as Ovarian Cancer and has the highest mortality rate among gynecological cancers^60^. miRNAs have been indicated to be promising biomarkers for Ovarian Cancer^60–62^. The dataset in this study included 114 known associations between miRNAs and Ovarian Neoplams. We performed the simulated experiment on Ovarian Neoplasms by removing all known associations related to Ovarian Neoplams and making them to be unknown. The simulated result showed that in top ten predicted miRNAs for Ovarian Neoplasms, three known associations have successfully been predicted and seven new associations have been reported. All of seven new predicted associations have been confirmed in other databases or literature. The top ten predicted associations for Ovarian Neoplasms in simulated experiment were shown in Table 7.

**Table 7:** Top 10 predicted ovarian neoplasms-associated miRNAs in the simulated experiment for predicting new disease-related miRNAs.

Rank	miRNA	Known before	Evidence(s)	Rank	miRNA	Known before	Evidence(s)
1	hsa-mir-1299	1	Known association	6	hsa-mir-26	0	PMID: 27158389
2	hsa-mir-224	1	Known association	7	hsa-mir-500b	0	dbDEMC V2.0
3	hsa-mir-1231	0	dbDEMC V2.0	8	hsa-mir-517c	0	PMID: 30687089
4	hsa-mir-1234	0	dbDEMC V2.0	9	hsa-mir-527	0	dbDEMC V2.0
5	hsa-mir-1301	0	dbDEMC V2.0	10	hsa-mir-92b	1	Known association

## Conclusion and discussions

Inferring potential miRNA–disease associations by integrating various types of prior information is a very challenging and meaningful work for disease-related researches. In this paper, we proposed a new method to infer miRNA–disease associations using improved random walk with restart and integrating multiple similarities (RWRMMDA) such as miRNA functional similarity, disease semantic similarity and network topological similarities of miRNA–disease association network. With Global LOOCV AUC (Area Under Roc Curve) and AUPR (Area Under Precision-Recall Curve) values of 0.9882 and 0.9066, respectively, and AUC and AUPR values of 0.9855 and 0.8642, respectively, under fivefold-cross-validation experiments, it illustrated that our proposed method achieved a reliable performance. In comparison with other related previous methods, it outperformed than NTSHMDA, PMFMDA, IMCMDA and MCLPMDA methods in both AUC and AUPR values. In case studies of Breast Neoplasms, Carcinoma Hepatocellular and Stomach Neoplasms diseases, it inferred 1, 12 and 7 new associations out of top 40 predicted associations, respectively. All of these new predicted associations have been confirmed in different databases or literatures. Therefore, our proposed method could be considered as a useful and meaningful tool to infer potential miRNA–disease associations.

There are some factors which contribute to the desirable performance of our proposed method as follows. Firstly, the known miRNA–disease associations which includes 5430 experimentally verified associations between 383 diseases and 495 miRNAs were gathered from the HMDD V2.0 database are reliable and they were used in many recent researches^4,14,27^. Secondly, both AUC and AUPR values of the proposed method were increased by using integrated similarities although it did not reduce the effect of sparsity data problem. Thirdly, the impact of sparsity data problem was reduced by performing a WKNKN algorithm as a pre-processing step to exclude unknown values in miRNA–disease association set based on their known neighbors. Therefore, the prediction performance becomes more informative. And finally, the most importance point is that the improved random walk with restart algorithm in our method was differed to common random walk with restart algorithms^18–20^. By supposing that a disease (miRNA) would have different relevant probabilities to each associated miRNA (disease), each miRNA–disease association was accredited different weight value in different heterogeneous network spaces which were built from integrating of multiple similarities. It would result in the trends to select actual miRNA–disease association couple with higher possibility when the extended random walk with restart algorithm was performed, from that prediction bias is limited.

Although our proposed approach achieves a reliable prediction performance and it could infer new disease-related miRNAs as indicated in the simulated experiments’ results of Lung Neoplasms and Ovarian Neoplasms in predicting new disease-related miRNAs section. However, subjectively choosing a new disease to perform simulated experiments by removing all its known associations can cause the bias in prediction. Therefore, it requires to do further researches or integrate more biological information to increase the reliability of prediction in case of new diseases or new miRNAs.

## Data Availability

The datasets were curated from public databases, HMDD V2.0 database (https://www.cuilab.cn/hmdd/) and MeSH descriptors (http://www.ncbi.nlm.nih.gov/). The processed data along with codes are available upon request.

## Abbreviations

AUC: Area Under ROC Curve
AUPR: Area Under Precision-Recall Curve
dbDEMC V2.0: Database of differentially expressed miRNAs in human cancers, version 2.0.
FN: False negative
FP: False positive
FPR: False positive rate
TP: True positive
TPR: True positive rate
miRNA: MicroRNA
mirCancer: MicroRNA Cancer Association Database
HCC: Hepatocellular carcinoma
WKNKN: Weighted K-nearest known neighbors
